# Sex-specific associations between the triglyceride-glucose index and new-onset hypertension in a hospital employee cohort: evidence from longitudinal annual health examinations

**DOI:** 10.3389/fendo.2025.1636890

**Published:** 2025-08-08

**Authors:** Ruixue Sun, Jianze Cai, Shaorong Yan, Jue Qian, Cheng Fu, Yuzhan Lin

**Affiliations:** ^1^ Department of Clinical Laboratory, The Third Affiliated Hospital of Wenzhou Medical University, Ruian, Zhejiang, China; ^2^ Hospital Infection Management Department (Public Health Department), The Third Affiliated Hospital of Wenzhou Medical University, Ruian, Zhejiang,, China; ^3^ Information Department, Ruian Traditional Chinese Medicine Hospital, Ruian, Zhejiang, China; ^4^ Department of Clinical Laboratory, Ruian Traditional Chinese Medicine Hospital, Ruian, Zhejiang, China

**Keywords:** TyG index, new onset hypertension, sex differences, insulin resistance, cohort study

## Abstract

**Background:**

The triglyceride-glucose (TyG) index is a surrogate marker of insulin resistance and has been associated with incident hypertension. However, evidence regarding sex-specific differences in this association remains limited. This study aimed to investigate whether sex modifies the association between the TyG index and incident hypertension in the general population.

**Methods:**

We conducted a retrospective cohort study involving 3,465 employees who underwent annual health check-ups in 2021 at the Third Affiliated Hospital of Wenzhou Medical University, with follow-up until December 2024. Participants with hypertension at baseline were excluded. The TyG index was calculated as ln [fasting triglycerides (mg/dL) × fasting glucose (mg/dL)/2]. Cox proportional hazards models and restricted cubic spline (RCS) analyses were used to evaluate the association between TyG index and incident hypertension across sex-specific subgroups. Sensitivity analyses tested robustness.

**Results:**

The incidence of hypertension increased across TyG quartiles in both sexes (p < 0.01). In women, the highest TyG quartile was associated with a significantly higher hypertension risk (HR = 1.82, 95% CI: 1.06–3.13). In men, the association was attenuated after adjustment. The results of the RCS analysis revealed that when TyG levels were high, the risk of hypertension was greater in men than in women. This conclusion was partially validated by the findings from the sensitivity analyses.

**Conclusions:**

In this retrospective cohort study based on annual health check-up data from hospital employees, we found that the TyG index may be positively associated with the risk of new-onset hypertension, with differences observed between sexes. Further research is needed to validate these findings and address potential confounding and concerns about generalizability.

## Introduction

Hypertension is one of the most widespread chronic conditions globally and remains a leading contributor to cardiovascular morbidity and mortality (1). According to the World Health Organization, approximately 1.28 billion adults aged 30 to 79 had hypertension in 2019, with fewer than one in five achieving adequate blood pressure control (2). In China, the prevalence of hypertension among community-dwelling adults aged 35 to 75 has reached 44.7% (age- and sex-standardized rate: 37.2%), affecting nearly half of the adult population, with a rising trend (3). More concerningly, recent epidemiological studies have reported an increasing trend in early-onset hypertension, with rising detection rates among young adults and the working population (4, 5). The rising incidence of hypertension among younger individuals highlights the importance of early detection and intervention during the prehypertensive or normotensive stages to prevent irreversible vascular damage and reduce long-term cardiovascular risk. Therefore, research on new-onset hypertension, particularly the early identification of high-risk individuals, is urgently needed.

Insulin resistance (IR) is considered both a pathogenic factor and an early predictor of hypertension (6). However, the clinical application of gold-standard methods, such as the hyperinsulinemic-euglycemic clamp test, is limited due to their complexity and invasiveness (7). The triglyceride-glucose (TyG) index, derived from fasting triglyceride and fasting glucose levels, has emerged as a simple and reliable surrogate marker of IR (8). Previous studies have shown that the TyG index is associated with elevated blood pressure and the development of hypertension. Individuals with higher TyG levels have a 1.51-fold increased risk of developing hypertension compared to those with lower levels (2). Additionally, longitudinal increases in TyG levels are also linked to elevated blood pressure and incident hypertension (9).

However, evidence regarding sex-specific differences in the association between the TyG index and new-onset hypertension remains limited, and findings across studies are inconsistent, with some reporting no sex differences (10–13) and one study suggesting the presence of differences (14). Moreover, these studies either conducted subgroup analyses using generalized linear models (10–13), or observed such differences only in populations aged over 40 years (14). Therefore, this study aims to examine sex-specific differences in the association between the TyG index and new-onset hypertension using longitudinal data from annual health check-ups of employees at the Health Examination Center of the Third Affiliated Hospital of Wenzhou Medical University.

## Methods

### Study population and ethics

Baseline was defined as the period from January 1 to December 31, 2021, during which all employees of the Third Affiliated Hospital of Wenzhou Medical University (Ruian People’s Hospital) underwent annual health examinations. All participants completed baseline questionnaires, physical examinations, and blood tests. A total of 3,465 individuals participated in the annual health check-ups in 2021. Participants were followed longitudinally from baseline in 2021 through December 31, 2024.

The exclusion criteria were as follows (1): participants without blood test data at baseline (2); those with pre-existing hypertension at baseline; and (3) those with unclear hypertension status during follow-up.

This study was approved by the Ethics Committee of the Third Affiliated Hospital of Wenzhou Medical University. Because the study was retrospective and the data were anonymized, the ethics committee waived written informed consent (Ethics approval number, YJ2025062).

### Definition of hypertension and new-onset hypertension

Hypertension was defined as meeting at least one of the following criteria (1): systolic blood pressure (SBP) ≥140 mmHg or diastolic blood pressure (DBP) ≥90 mmHg (2); use of antihypertensive medication; or (3) self-reported physician diagnosis of hypertension (15).

New-onset hypertension was defined as the development of hypertension during follow-up among participants who were normotensive at baseline. The occurrence of hypertension during follow-up was determined based on blood pressure measurements, medication records, and self-reported diagnoses. At each follow-up visit, the diagnosis of hypertension was based on the same three criteria described above. The date of blood pressure measurement, medication initiation, or self-report was recorded and used to calculate follow-up time. For participants who did not develop hypertension during follow-up, the follow-up duration was calculated as the time between baseline and the last available follow-up visit (9).

### Calculation of TyG index

The TyG index was calculated using the formula: TyG index = ln [fasting triglycerides (mg/dL) × fasting glucose (mg/dL)/2]

### Collection and definition of covariates

Baseline data were collected through comprehensive health examinations and included the following components: Demographic information included age, sex, marital status, family history of hypertension or diabetes, and department affiliation. Physical examination data included height, weight, body mass index (BMI), waist circumference, systolic blood pressure (SBP), and diastolic blood pressure (DBP). Laboratory results included complete blood count and biochemical tests, such as fasting plasma glucose, triglycerides (TG), total cholesterol (TC), high-density lipoprotein cholesterol (HDL-C), and low-density lipoprotein cholesterol (LDL-C).

Blood pressure was measured using an automated electronic sphygmomanometer after participants had rested in a seated position for at least 5 minutes. Personal and family histories of hypertension and diabetes were obtained via standardized questionnaires. Medication history was obtained from the hospital’s Health Information System (HIS); participants with records of antihypertensive drug use were considered to have received antihypertensive treatment. Marital status was self-reported and categorized as married or unmarried. Departments were classified according to their functional nature into clinical, medical technology, administrative, pharmacy, and retired groups. BMI was calculated as weight in kilograms divided by height in meters squared (kg/m²). Biochemical measurements were performed using fasting venous blood samples collected in yellow-top vacuum tubes after an overnight fast of at least 8 hours. Whole blood samples were centrifuged within 2 hours at 4°C using a refrigerated centrifuge at 1000–1200 ×g for 10–15 minutes to separate the serum (16).

### Statistical analysis

All analyses were conducted using R software (version 4.2.2; http://www.R-project.org, The R Foundation) and EmpowerStats (version 4.2; http://www.empowerstats.com, X&Y Solutions, Inc.).

Continuous variables were expressed as mean ± standard deviation (SD) or median with interquartile range (IQR), depending on their distribution. Categorical variables were presented as frequencies and percentages. For comparisons of continuous variables across four groups, one-way analysis of variance (ANOVA) was used if the data were normally distributed; otherwise, the Kruskal–Wallis test was applied. Categorical variables were compared using the chi-square test; if the expected cell count was less than 10, Fisher’s exact test was used instead. Cox proportional hazards models were used to evaluate the association between the TyG index and the risk of new-onset hypertension. Covariates were selected based on previously published literature (9, 17), and multivariable models were constructed to adjust for potential confounding factors. Regression results were presented as unadjusted, partially adjusted, and fully adjusted models (18). Restricted cubic spline (RCS) curves were used to examine potential nonlinear associations between the TyG index and the risk of new-onset hypertension in the overall population as well as in sex-specific subgroups, with log(HR) plotted on the Y-axis (19). To verify the stability of the results, a sensitivity analysis was performed in which RCS curves were analyzed separately in people who were not retired, were of Han ethnicity, and were not diabetic at baseline.

## Results

### Baseline characteristics of participants

**Figure 1:**
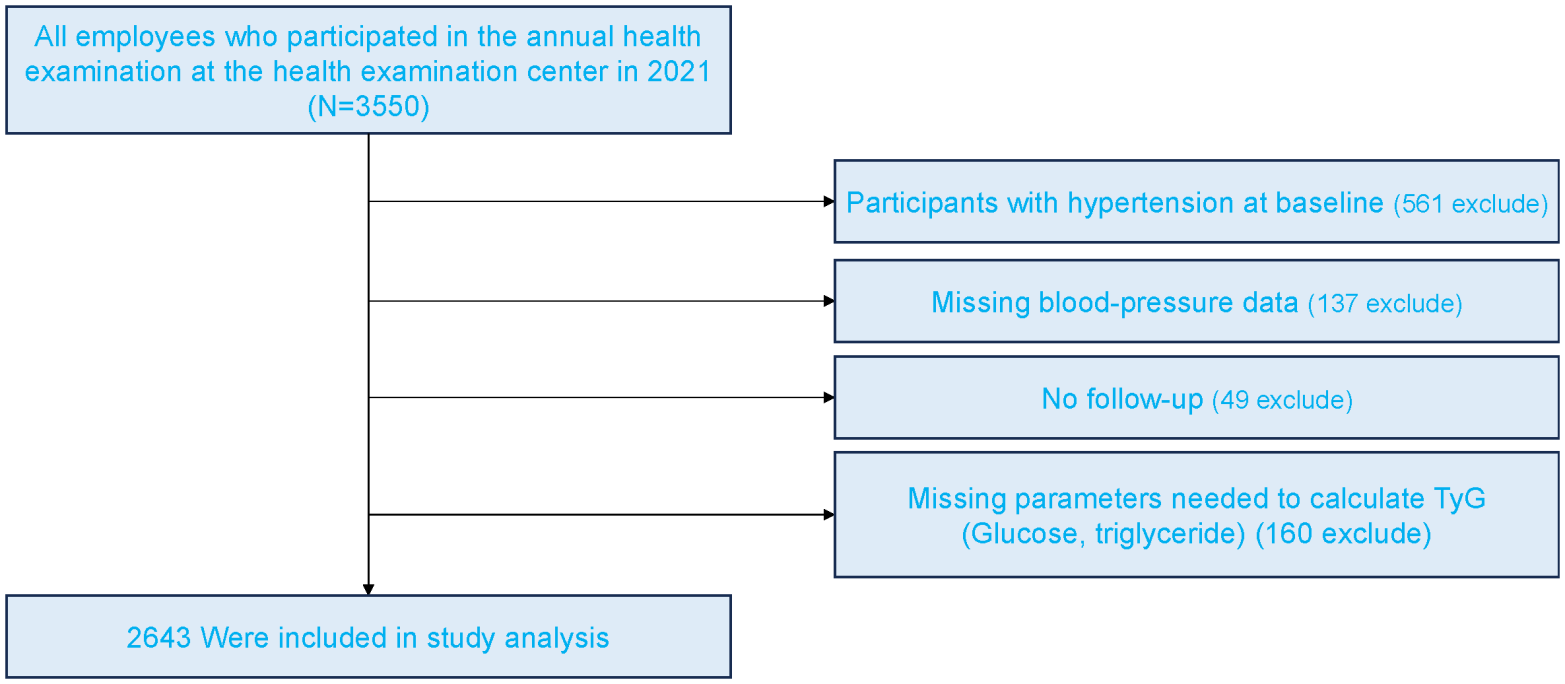
Flowchart of the study population.

**Figure 1** presents the flowchart of participant enrollment. Among the 3,465 employees who underwent annual health examinations in 2021, a total of 2,643 participants were included in the final analysis after applying exclusion criteria. Baseline characteristics stratified by TyG quartiles and sex are shown in **Table 1**. In both women and men, higher TyG levels were associated with older age, higher BMI, and elevated blood pressure (p < 0.001). Additionally, higher levels of glucose, triglycerides, uric acid, and liver enzymes were observed in participants with higher TyG levels (p < 0.001). The incidence of new-onset hypertension increased significantly across TyG quartiles, rising from 9.3% to 18.5% in women (p < 0.001) and from 11.8% to 29.2% in men (p = 0.007). As TyG levels increased, HDL-C levels decreased while LDL-C levels increased.

**Table 1:** Baseline characteristics of participants (N =2643).

TyG quartile	Female				P-value	Male				P-value
	Q1	Q2	Q3	Q4		Q1	Q2	Q3	Q4	
N	702	611	461	259		51	105	190	264	
Age, years	31.83 ± 7.47	33.00 ± 8.43	35.04 ± 10.00	39.08 ± 12.94	<0.001	32.49 ± 8.22	35.06 ± 10.02	35.49 ± 9.16	40.70 ± 11.32	<0.001
Marital status, %					<0.001					<0.001
Married	481 (68.52)	439 (71.85)	369 (80.04)	224 (86.49)		30 (58.82%)	77 (73.33%)	147 (77.78%)	234 (88.64%)	
Unmarried	221 (31.48)	172 (28.15)	92 (19.96)	35 (13.51)		21 (41.18%)	28 (26.67%)	42 (22.22%)	30 (11.36%)	
Retired, %					<0.001					0.346
No	693 (98.72)	594 (97.22)	438 (95.01)	217 (83.78)		50 (98.04%)	103 (98.10%)	187 (98.42%)	253 (95.83%)	
Yes	9 (1.28)	17 (2.78)	23 (4.99)	42 (16.22)		1 (1.96%)	2 (1.90%)	3 (1.58%)	11 (4.17%)	
Department, %					<0.001					0.033
CD	540 (76.92)	438 (71.69)	319 (69.20)	161 (62.16)		29 (56.86%)	57 (54.29%)	116 (61.05%)	145 (54.92%)	
AD	79 (11.25)	78 (12.77)	55 (11.93)	31 (11.97)		16 (31.37%)	20 (19.05%)	46 (24.21%)	70 (26.52%)	
Retired	8 (1.14)	17 (2.78)	23 (4.99)	42 (16.22)		1 (1.96%)	2 (1.90%)	3 (1.58%)	12 (4.55%)	
MTD	46 (6.55)	46 (7.53)	47 (10.20)	13 (5.02)		3 (5.88%)	16 (15.24%)	23 (12.11%)	29 (10.98%)	
PSD	29 (4.13)	32 (5.24)	17 (3.69)	12 (4.63)		2 (3.92%)	10 (9.52%)	2 (1.05%)	7 (2.65%)	
Race, %					0.356					0.678
Han	591 (98.17)	529 (99.44)	394 (99.24)	225 (99.56)		44 (100.00%)	84 (100.00%)	154 (98.72%)	206 (99.52%)	
Non-han	11 (1.83)	3 (0.56)	3 (0.76)	1 (0.44)		0 (0.00%)	0 (0.00%)	2 (1.28%)	1 (0.48%)	
DBP, mmHg	65.51 ± 8.13	66.16 ± 8.21	66.75 ± 7.96	69.01 ± 8.23	<0.001	71.26 ± 7.23	69.38 ± 8.99	71.21 ± 7.18	73.39 ± 7.83	<0.001
SBP, mmHg	108.28 ± 10.57	108.65 ± 10.95	109.57 ± 10.59	114.21 ± 11.59	<0.001	119.89 ± 10.07	119.00 ± 10.19	118.80 ± 9.54	121.16 ± 10.91	0.076
Pulse rate, beats/min (bpm)	80.81 ± 11.02	83.40 ± 12.10	83.03 ± 12.03	83.95 ± 11.27	<0.001	78.19 ± 14.23	74.22 ± 10.94	77.38 ± 10.64	78.09 ± 11.02	0.022
Height, cm	159.81 ± 5.26	159.39 ± 5.27	159.02 ± 5.66	158.59 ± 5.46	0.008	172.04 ± 5.37	172.26 ± 7.01	172.45 ± 5.89	170.75 ± 6.34	0.026
Weight, kg	51.84 ± 6.34	53.18 ± 7.51	54.38 ± 7.59	57.09 ± 8.69	<0.001	64.06 ± 9.72	67.15 ± 10.57	68.80 ± 9.06	70.22 ± 9.41	<0.001
BMI, kg/m²	20.29 ± 2.26	20.91 ± 2.61	21.51 ± 2.88	22.68 ± 3.09	<0.001	21.62 ± 2.98	22.58 ± 2.93	23.11 ± 2.62	24.06 ± 2.64	<0.001
WBC, 10^9^/L	5.42 ± 1.31	5.66 ± 1.45	5.86 ± 1.61	6.29 ± 1.78	<0.001	5.41 ± 1.22	5.91 ± 1.30	5.99 ± 1.24	6.48 ± 1.55	<0.001
NE, %	55.95 ± 7.85	56.04 ± 8.36	57.30 ± 8.74	58.21 ± 8.65	<0.001	53.63 ± 7.28	54.10 ± 7.80	54.19 ± 7.38	55.02 ± 7.40	0.366
AST, U/L	18.00 (16.00-21.00)	19.00 (16.00-22.00)	19.00 (16.00-22.00)	20.00 (17.00-25.00)	<0.001	21.00 (18.50-24.00)	22.00 (19.00-25.00)	23.00 (19.00-27.00)	24.00 (20.00-31.00)	<0.001
ALT, U/L	12.00 (10.00-16.00)	13.00 (10.50-18.00)	14.00 (11.00-19.00)	18.00 (13.00-26.00)	<0.001	19.00 (13.00-26.00)	21.00 (15.00-30.00)	24.00 (16.25-33.00)	31.00 (22.00-47.00)	<0.001
ALB, g/L	44.96 ± 2.06	45.10 ± 2.02	44.98 ± 2.31	45.11 ± 2.49	0.181	46.75 ± 2.11	45.87 ± 2.23	46.28 ± 2.05	46.38 ± 2.03	0.065
HDLC	1.53 ± 0.30	1.43 ± 0.27	1.33 ± 0.29	1.24 ± 0.32	<0.001	1.28 ± 0.22	1.23 ± 0.26	1.17 ± 0.25	1.07 ± 0.21	<0.001
LDLC	2.55 ± 0.73	2.76 ± 0.71	3.02 ± 0.79	3.14 ± 0.95	<0.001	2.84 ± 0.76	2.95 ± 0.71	3.21 ± 0.77	3.35 ± 0.95	<0.001
Glu, mmol/L	4.36 ± 0.33	4.51 ± 0.35	4.58 ± 0.41	4.82 ± 0.81	<0.001	4.30 ± 0.44	4.47 ± 0.49	4.54 ± 0.47	4.91 ± 1.07	<0.001
TG, mmol/L	0.62 ± 0.10	0.87 ± 0.10	1.20 ± 0.16	2.15 ± 0.97	<0.001	0.68 ± 0.09	0.90 ± 0.11	1.26 ± 0.18	2.34 ± 0.96	<0.001
UA, μmol/L	280.64 ± 60.82	286.37 ± 58.14	294.08 ± 65.57	318.10 ± 78.35	<0.001	391.92 ± 64.96	396.72 ± 73.74	412.68 ± 80.13	439.34 ± 86.42	<0.001
History of diabetes, %					0.036					0.012
No	695 (99.00)	606 (99.18)	452 (98.05)	251 (96.91)		51 (100.00%)	102 (97.14%)	187 (98.42%)	246 (93.18%)	
Yes	7 (1.00)	5 (0.82)	9 (1.95)	8 (3.09)		0 (0.00%)	3 (2.86%)	3 (1.58%)	18 (6.82%)	
Family history of hypertension, %					0.092					0.908
No	323 (96.42)	284 (95.30)	232 (97.07)	109 (91.60)		32 (96.97%)	53 (98.15%)	95 (95.96%)	134 (96.40%)	
Yes	12 (3.58)	14 (4.70)	7 (2.93)	10 (8.40)		1 (3.03%)	1 (1.85%)	4 (4.04%)	5 (3.60%)	
New-onset hypertension, %					<0.001					0.007
No	637 (90.74)	540 (88.38)	415 (90.02)	211 (81.47)		45 (88.24%)	85 (80.95%)	154 (81.05%)	187 (70.83%)	
Yes	65 (9.26)	71 (11.62)	46 (9.98)	48 (18.53)		6 (11.76%)	20 (19.05%)	36 (18.95%)	77 (29.17%)	

Baseline characteristics are presented separately by sex. Within each sex, participants were categorized into four TyG quartiles, and comparisons across quartiles were conducted using one-way ANOVA for normally distributed continuous variables, Kruskal–Wallis test for skewed continuous variables, and chi-square or Fisher’s exact test for categorical variables. *Post hoc* pairwise comparisons were not performed, as the purpose was descriptive comparison of baseline characteristics.

BMI, body mass index; WBC, white blood cell count; NE, neutrophil percentage; AST, aspartate aminotransferase; ALT, alanine aminotransferase; ALB, serum albumin; DBP, diastolic blood pressure; SBP, systolic blood pressure; Glu, fasting blood glucose; TG, triglycerides; UA, uric acid; TyG, triglyceride-glucose index; CD, clinical department; AD, administrative department; MTD, medical technology department; PSD, pharmaceutical sciences department; HDL-C, high density lipoprotein cholesterol; LDL-C, low-density lipoprotein cholesterol.

### Association of TyG index with incident hypertension in sex-specific subgroups

**Table 2** presents the results of the Cox regression analysis. In the unadjusted model, higher TyG index levels were associated with an increased risk of new-onset hypertension in both men and women. Among women, the highest TyG quartile (Q4) was significantly associated with an increased risk of hypertension compared to the lowest quartile (Q1) in the unadjusted model (HR = 2.16, 95% CI: 1.49–3.14, P < 0.0001), and the association remained significant after full adjustment (HR = 1.82, 95% CI: 1.06–3.13, P = 0.0307). In men, a significant association was also observed in the unadjusted model (Q4 vs. Q1: HR = 3.05, 95% CI: 1.33–7.01, P = 0.0085), but it was no longer significant after full adjustment. In the overall population, a significant positive association between the TyG index and hypertension was observed in the unadjusted model, but the association was not significant in the fully adjusted model.

**Table 2:** Associations between TyG index and new-onset hypertension.

Exposure	Non-adjusted (HR, 95%CI, P)	Adjust I (HR, 95%CI, P)	Adjust II (HR,95%CI, P)
In female			
TyG continuous	1.44 (1.10, 1.87) 0.0070	1.16 (0.83, 1.62) 0.3738	1.15 (0.78, 1.69) 0.4920
TyG quartile			
Q1	1.0	1.0	1.0
Q2	1.28 (0.91, 1.79) 0.1494	1.17 (0.78, 1.77) 0.4443	1.26 (0.83, 1.90) 0.2788
Q3	1.11 (0.76, 1.62) 0.5975	0.88 (0.55, 1.42) 0.6092	0.91 (0.55, 1.50) 0.7040
Q4	2.16 (1.49, 3.14) <0.0001	1.71 (1.07, 2.74) 0.0263	1.82 (1.06, 3.13) 0.0307
P for trend	0.0011	0.1256	0.1347
In male			
TyG continuous	2.01 (1.51, 2.67) <0.0001	1.20 (0.78, 1.83) 0.4064	1.07 (0.60, 1.93) 0.8081
TyG quartile			
Q1	1.0	1.0	1.0
Q2	1.72 (0.69, 4.28) 0.2462	1.49 (0.54, 4.16) 0.4432	1.62 (0.56, 4.70) 0.3707
Q3	1.71 (0.72, 4.05) 0.2264	0.93 (0.34, 2.52) 0.8879	0.84 (0.30, 2.33) 0.7357
Q4	3.05 (1.33, 7.01) 0.0085	1.45 (0.55, 3.81) 0.4535	1.32 (0.46, 3.79) 0.6092
P for trend	0.0004	0.5969	0.9780
In total			
TyG continuous	1.67 (1.38, 2.03) <0.0001	1.15 (0.89, 1.48) 0.2731	1.08 (0.79, 1.48) 0.6133
TyG quartile			
Q1	1.0	1.0	1.0
Q2	1.32 (0.96, 1.80) 0.0837	1.25 (0.86, 1.81) 0.2394	1.30 (0.89, 1.90) 0.1713
Q3	1.20 (0.87, 1.67) 0.2652	0.91 (0.60, 1.36) 0.6386	0.90 (0.59, 1.39) 0.6366
Q4	2.27 (1.66, 3.10) <0.0001	1.53 (1.02, 2.28) 0.0377	1.52 (0.96, 2.41) 0.0776
P for trend	<0.0001	0.1213	0.2561

Non-adjusted model adjust for: None

Adjust I model adjust for: Age; Race; Department; BMI.

Adjust II model adjust for: Age; Race; Department; BMI; Marital status; History of diabetes; WBC; NE; AST; ALT; ALB; HDL-C; LDL-C; UA.

BMI, body mass index; WBC, white blood cell count; NE, neutrophil percentage; AST, aspartate aminotransferase; ALT, alanine aminotransferase; ALB, serum albumin; TyG, triglyceride-glucose index; HDL-C, high density lipoprotein cholesterol; LDL-C, low-density lipoprotein cholesterol; UA, uric acid.

**Figure 2:**
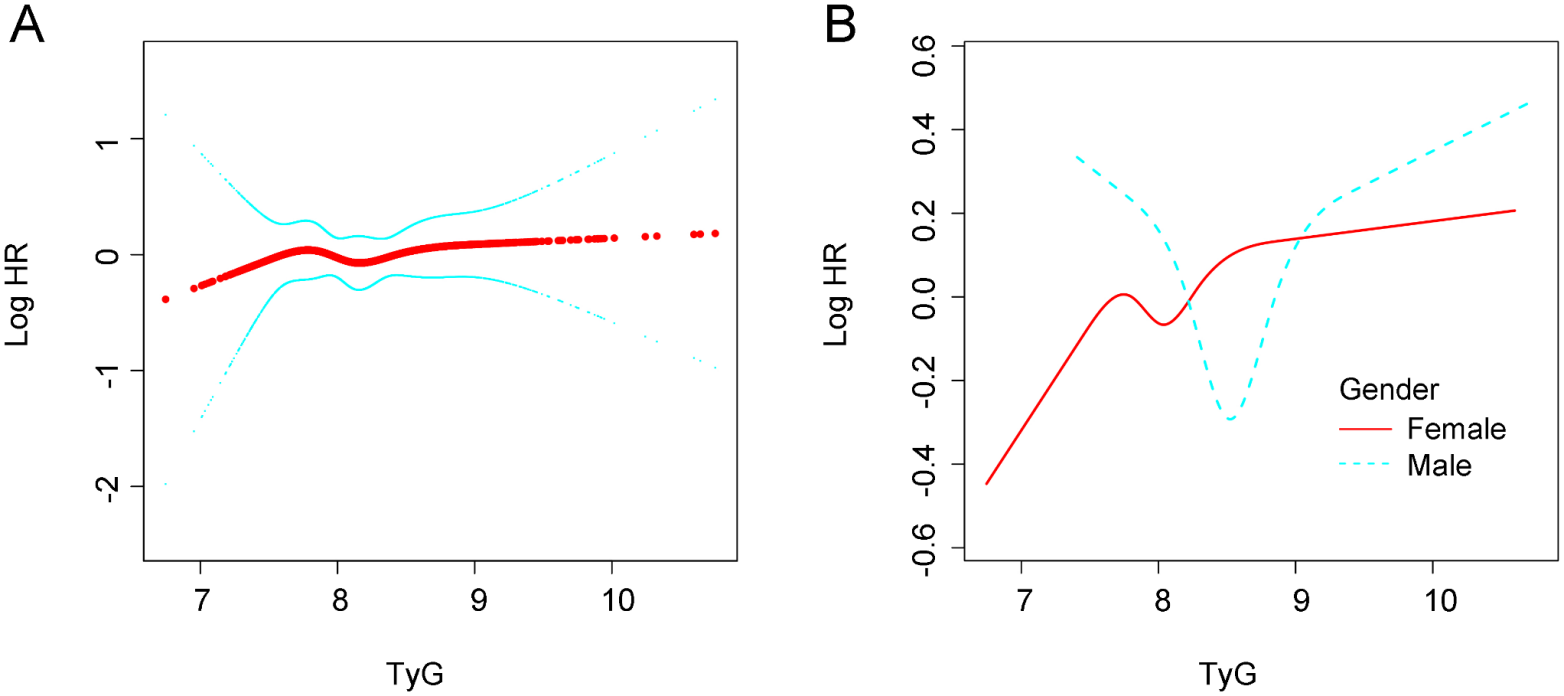
Association between TyG and new-onset hypertension. **(A)** in the general population. **(B)** In different gender groups. All analyses were adjusted for confounding factors including. Age; ARace; Department; BMI; Marital status; History of diabetes; WBC; NE; AST; ALT; ALB; HDL-C; LDL-C; UA. BMI, body mass index; WBC, white blood cell count; NE, neutrophil percentage; AST, aspartate aminotransferase; ALT, alane aminotransferase; ALB, serum neutrophil perecntage; AST, aspartate aminotransferase; ALT, alane aminotransferase; ALB, serum albumin; TyG, tryglyceride-fglucose index; HDL-C, High density liproprotein cholesterol; LDL-C, Low-density lipoprotein cholesterol; UA, uric acid.

**Figure 2** illustrates the association between the TyG index and the risk of new-onset hypertension using a RCS model. In the overall population, a nonlinear positive relationship was observed between the TyG index and the risk of new-onset hypertension. In **Figure 2B**, stratified analyses by sex showed that the risk of new-onset hypertension increased with rising TyG levels in both men and women. However, the risk of hypertension differed between sexes at different TyG levels, with men exhibiting a greater risk than women when the TyG index reached higher levels.

### Sensitivity analysis

**Figure 3:**
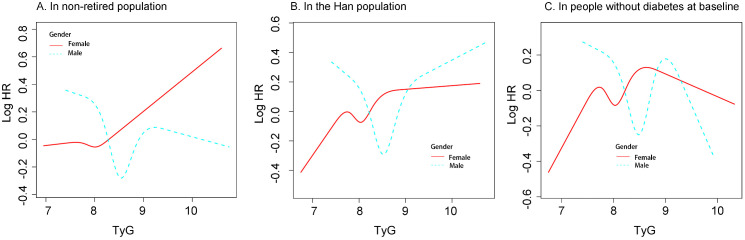
The associatioon between TyG and new-onset hypertension differed by gender in different population **(A)** In non-retired popultaion. **(B)** in the Han population. **(C)** In people without diabetes at baseline. Restricted cubic spline analysis was used to construct stratification curves, and all analyses were adjusted for confounding factors. Including: Age : Race; Department; BMI;Marital status; History of diabtes; WBC; NE; AST; ALT; ALB; HDL-C; LDL-C; UA (Excluding the variable of restricted poplulation) BMI, body mass index; WBC, white blood cell count; NE, Neutrophil percentage; AST, aspartate aminotransferase; ALT, alanine amonitransferase; ALB,serum albumin; TyG, triglyceride-glucose index; HDL-C, High density lipoprotein cholesterol; LDC-C, low density lipoprotein cholesterol; UA, Uric acid.

Stratified analyses using the RCS model were performed in the non-retired population, Han Chinese participants, and individuals without diabetes at baseline (**Figure 3**). In all these subgroups, sex differences were observed in the association between TyG levels and new-onset hypertension. Among Han participants and those without baseline diabetes, women had a lower incidence of hypertension than men when TyG levels were high. However, this pattern was not observed in the non-retired population.

## Discussion

In this retrospective cohort study based on annual health check-up data of hospital employees, we found that higher TyG index levels were significantly associated with an increased risk of new-onset hypertension, and this association differed by sex. To our knowledge, this is the first study to investigate sex-specific differences in the association between the TyG index and incident hypertension.

Previous studies have also confirmed a positive association between the TyG index and new-onset hypertension. For example, Wang et al. (9) reported that baseline TyG levels were positively associated with new-onset hypertension. Each one-unit increase in TyG was associated with a 21% higher risk of hypertension (HR = 1.21, 95% CI: 1.13–1.29). Similarly, a 9-year cohort study by Zheng et al. (20) reported consistent findings, with participants in the highest TyG quartile having a significantly higher risk of hypertension compared to those in the lowest quartile (Q4 vs. Q1: HR = 1.53, 95% CI: 1.07–2.19). In addition, Gao et al. (11) and Liu et al. (10) analyzed two large nationwide datasets with representative Chinese populations—the CHNS and CHARLS—and reported similar results. Similar associations were also observed in studies conducted among Singaporean (21) and Spanish (13) populations. In the two studies based on nationwide Chinese population data, generalized linear models were used for subgroup analyses by sex. No significant sex differences were observed (CHARLS: male HR = 1.17, female HR = 1.20; CHNS: male HR = 1.27, female HR = 1.35). In our study, Cox regression analysis showed that the HR for women (1.18) was slightly higher than that for men (1.01), which is consistent with previous findings. Using RCS, we found a sex difference in the association between TyG levels and new-onset hypertension. Among individuals with higher TyG levels, women had a lower risk of hypertension compared to men. Our study also collected information on participants’ departments and adjusted for this variable in the analysis. This is a strength of our study, as in Chinese hospitals, different departments are often associated with distinct work patterns and lifestyle behaviors (22, 23). A recent meta-analysis concluded that the association between TyG and new-onset hypertension is not affected by sex (2). This conclusion is controversial compared to our findings. Since our data are derived from a single-center study, additional evidence is needed to further explore sex differences and support our observations.

In the sensitivity analyses, we found significant differences in the risk of new-onset hypertension between men and women as TyG levels increased across different subpopulations. Among Han participants and those without diabetes at baseline, women had a lower risk of developing hypertension than men at higher TyG levels. However, this pattern was not observed in the non-retired population. Considering that our study population consisted of hospital employees from various clinical and administrative departments, female healthcare workers may experience greater occupational stress, particularly nurses. Factors such as night shifts and heavy workloads may increase the risk of metabolic disorders and elevated blood pressure in this group (24). In addition, women often bear additional family responsibilities in society (25), which may partially explain our findings. However, further studies are needed to support this hypothesis.

Several potential pathophysiological mechanisms may explain the association between the TyG index and hypertension. The TyG index is widely recognized as a surrogate marker of insulin resistance (26), which influences blood pressure through multiple pathways. For example, IR can lead to endothelial dysfunction by reducing nitric oxide production, which impairs vasodilation and increases peripheral vascular resistance (27). Additionally, IR activates the sympathetic nervous system through both central and peripheral mechanisms, leading to increased heart rate and vasoconstriction (28). It also promotes renal sodium reabsorption, resulting in fluid retention and increased blood volume (29). Moreover, individuals with elevated TyG levels often exhibit chronic low-grade inflammation and sustained oxidative stress, which contribute to atherosclerosis and vascular remodeling (30), further increasing blood pressure.

In our study, the risk of new-onset hypertension tended to stabilize in women with higher TyG levels compared to men. We speculate that this phenomenon may be attributed to the protective effects of estrogen on lipid metabolism, insulin resistance, and cardiovascular as well as blood pressure regulation. Estrogen has been shown in multiple studies to enhance insulin sensitivity by promoting glucose uptake in peripheral tissues and suppressing hepatic gluconeogenesis, thereby lowering blood glucose levels (31). It also mobilizes systemic fat stores and reduces visceral fat accumulation (32). In addition, estrogen downregulates hepatic angiotensinogen synthesis, thereby inhibiting the renin–angiotensin–aldosterone system, reducing vasoconstriction and sodium retention (33). Estrogen also inhibits both central and peripheral sympathetic nervous system activity, leading to reduced norepinephrine release and vascular tone, which ultimately lowers blood pressure (34). Furthermore, estrogen enhances endothelial function and vasodilation by activating endothelial nitric oxide synthase and promoting nitric oxide production (35). Beyond blood pressure regulation, estrogen also exerts significant anti-inflammatory and antioxidant effects. Estrogen suppresses the expression of pro-inflammatory cytokines such as tumor necrosis factor-α, interleukin-6, and C-reactive protein (36), while upregulating anti-inflammatory cytokines like interleukin-10 (37), thereby modulating and reducing systemic inflammation.

This study also has several limitations. First, the data were obtained from a single center and included only hospital employees undergoing annual health examinations, which may limit the generalizability of our findings to other populations. Second, although we adjusted for multiple confounders as much as possible, unmeasured confounding remains due to the retrospective nature of this study. Variables such as smoking, alcohol consumption, sleep apnea, sleep quality, dietary habits, and physical activity were not collected. Therefore, we collected other variables to partially account for relevant confounding factors. For example, we collected information on participants’ clinical department to serve as a proxy for occupational stress. However, residual confounding cannot be completely eliminated. Third, due to the observational nature of this study, we can only establish an association between the TyG index and new-onset hypertension, rather than causality. Fourth, we analyzed only baseline TyG levels and did not assess the potential impact of longitudinal changes in TyG on hypertension risk. Further studies are warranted to explore this issue.

## Conclusions

In this retrospective cohort study based on annual health check-up data from hospital employees, we found that the TyG index may be positively associated with the risk of new-onset hypertension, and this association may be nonlinear. Moreover, our analysis suggested that at higher TyG levels, men had a greater risk of developing hypertension compared to women. Further studies are needed to address potential confounding and improve the generalizability of these findings.

## Data Availability

The raw data supporting the conclusions of this article will be made available by the authors, without undue reservation.
